# Application accuracy of a frameless optical neuronavigation system as a guide for craniotomies in dogs

**DOI:** 10.1186/s13028-023-00720-y

**Published:** 2023-12-14

**Authors:** Sarah Gutmann, Miriam Heiderhoff, Robert Möbius, Tanja Siegel, Thomas Flegel

**Affiliations:** 1 Department for Small Animals, Faculty of Veterinary Medicine, Leipzig University, An den Tierkliniken 23, 04103 Leipzig, Germany; 2 Department of Neurosurgery, Faculty of Medicine, University Clinic of Leipzig, Leipzig, Germany

**Keywords:** Brain surgery, Canine medicine, Frameless neuronavigation, Neuronavigation device, Neurosurgery

## Abstract

**Background:**

Optical neuronavigation systems using infrared light to create a virtual reality image of the brain allow the surgeon to track instruments in real time. Due to the high vulnerability of the brain, neurosurgical interventions must be performed with a high precision. The aim of the experimental cadaveric study was to determine the application accuracy of a frameless optical neuronavigation system as guide for craniotomies by determining the target point deviation of predefined target points at the skull surface in the area of access to the cerebrum, cerebellum and the pituitary fossa. On each of the five canine cadaver heads ten target points were marked in a preoperative computed tomography (CT) scan. These target points were found on the cadaver skulls using the optical neuronavigation system. Then a small drill hole (1.5 mm) was drilled at these points. Subsequently, another CT scan was made. Both CT data sets were fused into the neuronavigation software, and the actual target point coordinates were identified. The target point deviation was determined as the difference between the planned and drilled target point coordinates. The calculated deviation was compared between two observers.

**Results:**

The analysis of the target point accuracies of all dogs in both observers taken together showed a median target point deviation of 1.57 mm (range: 0.42 to 5.14 mm). No significant differences were found between the observers or the different areas of target regions.

**Conclusion:**

The application accuracy of the described system is similar to the accuracy of other optical neuronavigation systems previously described in veterinary medicine, in which mean values of 1.79 to 4.3 mm and median target point deviations of 0.79 to 3.53 mm were determined.

## Background

Modern neuronavigation systems are becoming increasingly important in small animal medicine for neurosurgical interventions, such as brain biopsies, removal of brain tumors or other brain surgery indications like the insertion of electrodes for deep brain stimulation [1–8]. Recently there have also been initial efforts to use neuronavigation systems in the field of spinal surgery in small animals [9].

Neuronavigation systems create a virtual reality image of the brain allowing the surgeon to track instruments in real time [10]. This improves the surgeon’s three-dimensional orientation during the operation and should lead to a reduction in complications. They are helpful in determining surgical access, which can shorten surgery time and help keeping the approach as small as possible. There are two different neuronavigation systems currently in use: optical and electromagnetic neuronavigation systems [10]. Optical neuronavigation systems consist of a dual camera system emitting infrared light and reflective markers (spheres) identifying the patient as well as the instruments being used. Both cameras emit light which is reflected by those spheres and registered by the cameras again. Electromagnetic neuronavigation systems use the deformation of a magnetic field emitted by the magnetic sensor system for determination of the position of the skull and instruments. A software calculates the position of the instruments in relation to the patient`s head based on markers that can be identified on magnetic resonance (MR) or computed tomography (CT) images taken prior to the procedure as well as on the real patient during surgery. The matching of the surgical situs and real instruments into the virtual coordinate system of the preoperatively acquired imaging data sets of the patient is performed via an image-to-patient registration process prior to the surgery.

In the study presented here a new optical neuronavigation system of the company STORZ was used, which was originally designed for human ENT (ear, nose and throat) surgery. The setup and associated devices were adapted for clinical use in small animal neurosurgery. The aim of the cadaver study was to determine the application accuracy of the frameless optical neuronavigation system “NAV 1 pico” of the company STORZ for targeting predefined points on the skull surface for the surgical access to the cerebrum, the cerebellum, and the pituitary gland.

## Methods

**Fig. 1:**
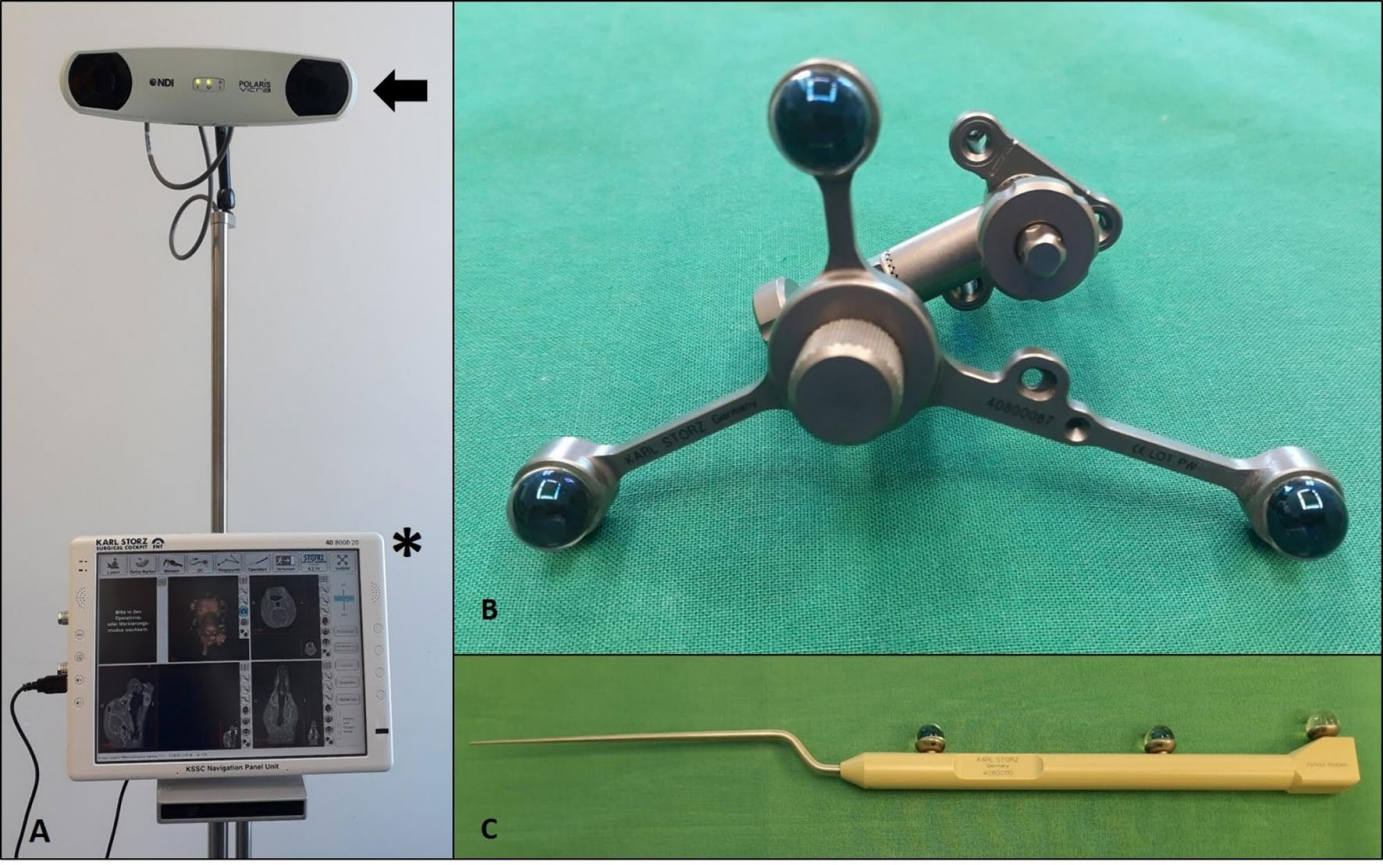
Components of the optical neuronavigation system “NAV 1 pico”. The frameless optical neuronavigation system “NAV 1 pico” (Karl STORZ, Tuttlingen, Deutschland) consists of a dual infrared camera system (**A**, black arrow), a special neuronavigation software (**A**, asterisk), a patient tracker with the reflective spheres (**B**) and the probe with the reflective elements (**C**)

The neuronavigation system “NAV 1 pico” (Karl STORZ, Tuttlingen, Germany) was used. The device consists of a dual camera system, emitting infrared light, a neuronavigation panel unit with a neuronavigation software (Karl STORZ navigation software, Tuttlingen, Germany), a patient tracker with three reflective spheres and the probe with three reflective elements (Fig. 1). The most important technical information of the used optical neuronavigation system is summarized in Table 1.

**Table 1:** Technical information of the frameless optical neuronavigation system “NAV 1 pico” (Karl STORZ, Tuttlingen, Germany)

Maximum measuring volume	0.35 m³
Technical 3D accuracy determined by the manufacturer	up to 0.25 mm (RMS)
Maximum measuring rate	20 Hz
Registration limit for surgical use	0.5 mm

RMS – root mean square

**Fig. 2:**
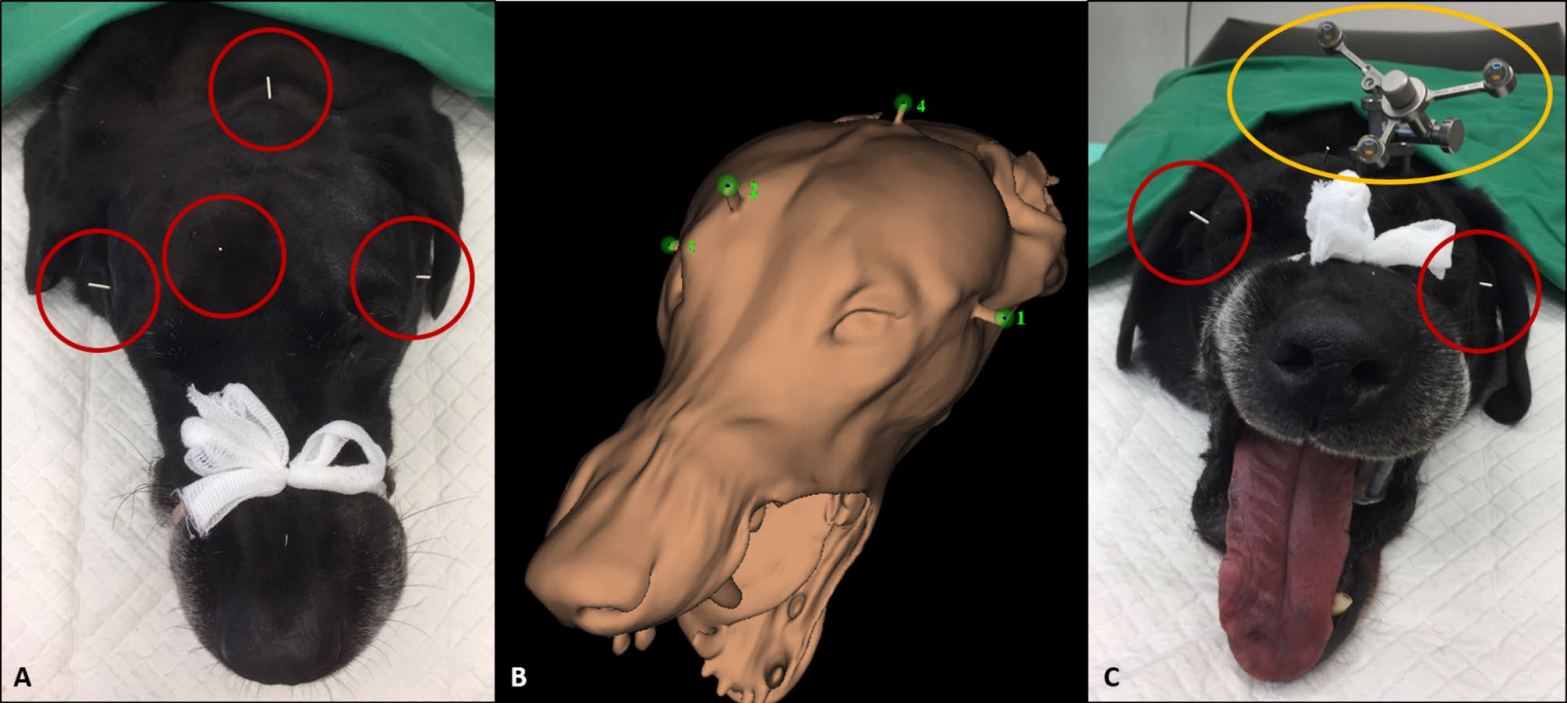
Illustration of important steps of the study on a canine cadaver head. (**A**) Canine cadaver head with the four pins for the registration process in place: on both sides of the zygomatic arch, paramedian over the frontal sinus and into the occipital protuberance (red circles). After the placement of the four pins a CT scan of the skull was made. (**B**) 3D reconstruction of a cadaver head with the four K-wire pins in position. On each tip of the pins, a marker point for the registration process was set (green dots). (**C**) Canine cadaver head after placement of the patient tracker with the three reflective spheres paramedian over the frontal sinus (yellow ellipse). The two pins on both sides of the zygomatic arch are also visible (red circles). The patient tracker and the four pins were needed for the registration process of the skull. Thereby, the real operation situs was matched with the preoperative CT images of the skull

For the study five canine cadavers were used. All dogs were euthanized for reasons unrelated to the study. The canine cadaver heads were prepared as follows: First the hair in the region of the neurocranium was clipped. Then four K-wire pins (1.4 mm) were placed at the following localizations: both sides of the zygomatic arches, paramedian over the frontal sinus and into the occipital protuberance (Fig. 2A). The K-wire pins are necessary for the registration process of the neuronavigation system later. Afterwards a mouth gag was placed to keep the mouth open. Then a CT scan (Philips IQon spectral CT, Philips Healthcare, Hamburg, Germany) of the whole skull from the nasal plenum to the neck, including the pins, was made using the following scanning parameters: slice thickness: 1 mm; reconstruction filter: y-sharp (yc); voxel: 1 mm x 0.3 mm x 0.3 mm; matrix: 512 × 512 pixel; collimation 64 × 0.625 mm; field of view (FOV) 196 × 196 mm; increment: 0.5 mm.

The CT images were imported into the neuronavigation software. Based on the CT dataset, the neuronavigation software reconstructed a surface rendering of the canine skulls. Ten target points (X, Y, Z- coordinates) in each cadaver head in different regions were marked in the imported scan. In this way a total of 50 target point at the skull surface of all five cadaver heads were planned, of which 32 target points were set in the region of access to the cerebrum (frontal or parietal bone), 8 target points in the region of access to the cerebellum (occipital bone) and 10 target points in the region of access to the pituitary fossa (basisphenoid bone).

Four digital marker points were set in the surface rendering of the neuronavigation software for the registration process, with one marker point placed on each tip of every K-wire pin (Fig. 2B). Then the patient tracker with the three reflective spheres was attached paramedian over the frontal sinus using three small self-cutting titanium screws (Fig. 2C). The registration process for matching the surgical situs with the pre-operatively image datasets was made by touching a small preset recess on the patient tracker and then the tip of each pin in a predefined order with the probe while reflecting the infrared light through the reflective spheres of the tracker and the probe.

**Fig. 3:**
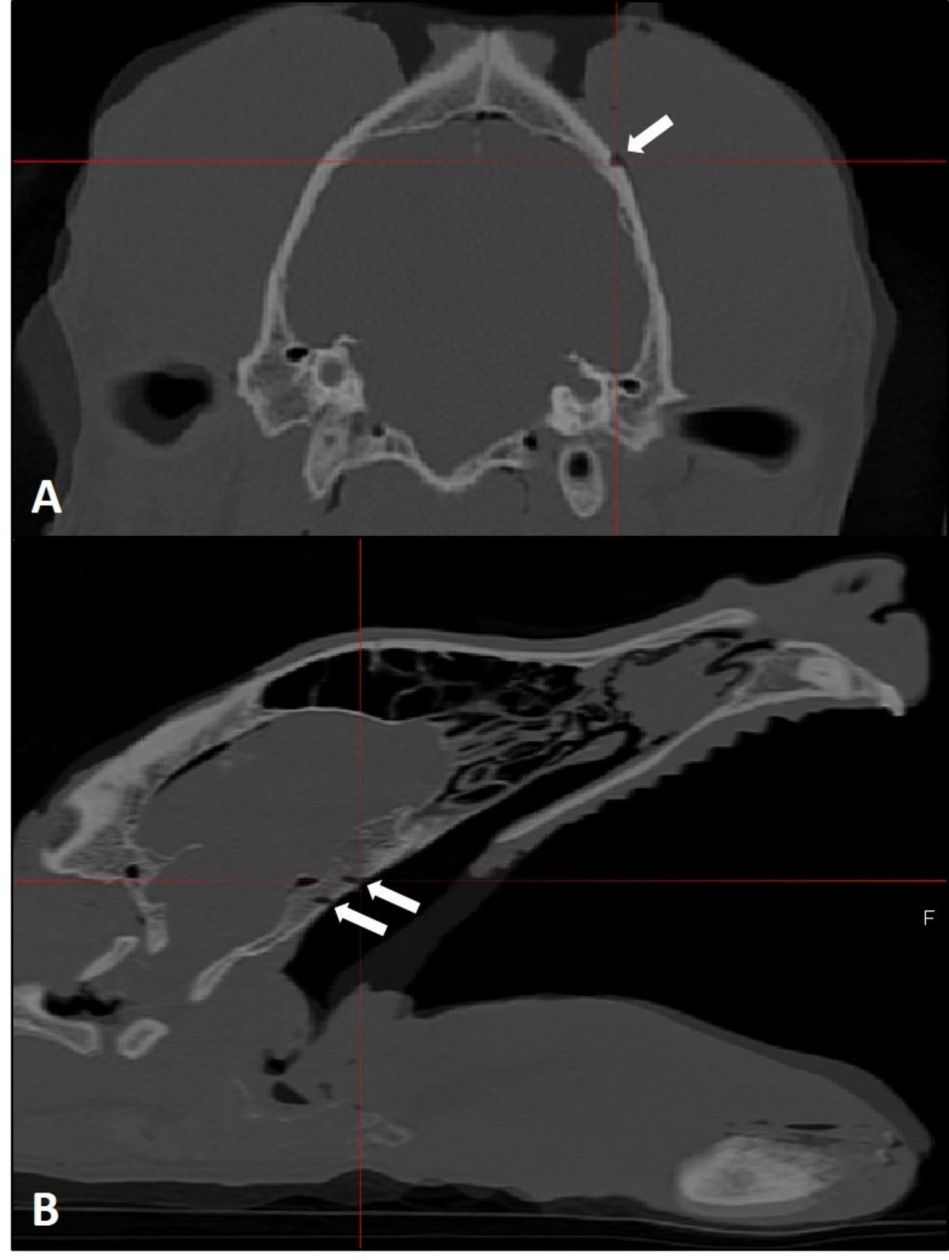
Fused pre- and postoperative CT datasets of a canine skull. (**A**) Transverse CT image of a canine skull. The white arrow mark one of the drill holes in the region for the access to the cerebrum. (**B**) Sagittal CT image of a canine skull. The two white arrows mark two drill holes in the region for the access to the pituitary gland

Subsequently, the planned target points were identified with the probe and an access to the skull surface in the regions was made. The target points were marked with a permanent marker on the skull bone. Then a 1.5 mm drill hole was drilled at each of the ten target points of every cadaver head. Afterwards, a second CT scan of the skull was made with the same scanning parameters as already described. The second CT dataset was imported in the neuronavigation software and then both CT sets were fused in one coordinate system. Afterwards, two observers independently analyzed the target points after fusion of both datasets. Each observer determined the actual coordinates (X`, Y`, Z`) of the center of each drill hole in each cadaver (Fig. 3A and B). The target point deviation was then calculated using the formula:$D =\sqrt{{\left(x-{x}^{{\prime }}\right)}^{2}+{\left(y-{y}^{{\prime }}\right)}^{2}+{\left(z-{z}^{{\prime }}\right)}^{2}}$ between the predefined target points (X, Y, Z) and the actual drilled target points (X`, Y`, Z`).

## Statistics

Statistical comparison of the data was performed using Microsoft Excel (Microsoft Excel, version 2013, Redmond, Washington, USA) and SPSS software (SPSS software, version 24.0, IBM, Armonk, New York, USA). The data were tested for normal distribution using the Kolmogorov-Smirnov test. Data were presented as the mean with the SD or the median with the range based on their normality. The comparison between the groups and observers was performed using the nonparametric Mann-Whitney U Test and the Kruskal-Wallis-test. P-values of 0.05 or less were considered statistically significant.

## Results

Five canine cadaver heads of the following breeds were used: Labrador retriever (n = 2), German shepherd (n = 1), Dachshund (n = 1) and Siberian Husky (n = 1). The median body weight was 30.2 kg (range: 10.0 to 43.0 kg).

In the region of the access to the cerebrum (frontal and parietal bone) 32 target points were planned and then drilled using the neuronavigation system, in the region of access to the cerebellum (occipital bone) 8 and in the region of access to the pituitary fossa (basisphenoid bone) 10 target points were planned and drilled. The coordinates of 50 planned and then drilled target points were read out from two observers. Therefore, a total of 100 target point coordinates were analyzed.

**Fig. 4:**
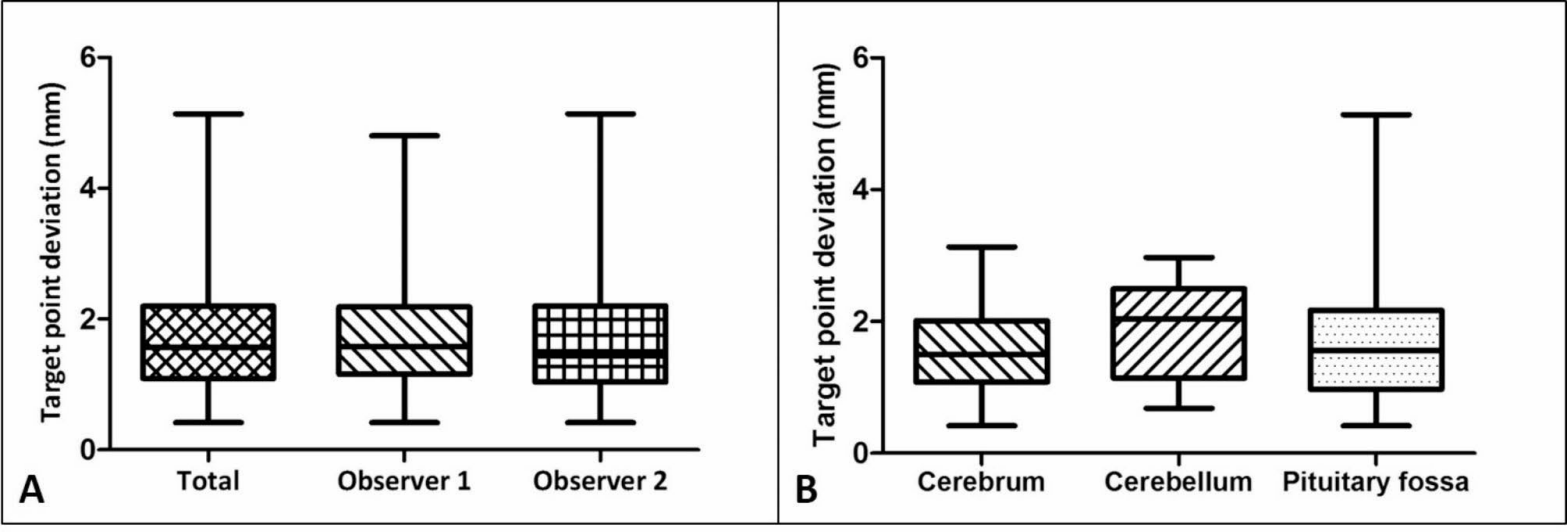
Graphic illustration of the target point deviation. Box and whisker plots displaying the target point deviation of all target points in mm using the frameless optical neuronavigation system. The whiskers represent minimum and maximum values, the black line inside each box the median value and lower and upper box boundaries 25th and 75th percentiles, respectively. (**A**) Target point deviation of all 50 predetermined target points read out by two observers (total, n = 100) and for both observers separately (n = 50). (**B**) Target point deviation divided in groups depending on affiliation of access to the cerebrum (frontal bone and parietal bone, n = 64), cerebellum (occipital bone, n = 16) and pituitary fossa (basisphenoidal bone, n = 20)

The median target point error of all target points of all dogs evaluated by both observers was 1.57 mm (range: 0.42 to 5.14 mm, see Table 1). The median target point deviation of all target points in all dogs evaluated by observer 1 was 1.58 mm (range: 0.42 to 4.8 mm) and by observer 2 1.44 mm (range 0.42 to 5.14 mm) respectively. The analysis of the target point accuracies in the area of the access to the cerebrum of all dogs in both observers together showed a median target point deviation of 1.51 mm (range 0.42–3.13 mm), in the area of the cerebellum 2.04 mm (range 0.68–2.97 mm) and in the area of the pituitary gland 1.56 mm (range 0.42–5.14 mm). No significant difference in target point deviations could be demonstrated between the observers (P = 0.775) or between the individual groups of different access to the brain (P = 0.423). All median values with minimum and maximum, as well as mean values with standard deviation of the target point deviations of both investigators and the individual groups are shown in the Table 2, as well as in Fig. 4.

**Table 2:** Target point deviation of the drill holes in mm

	Total (n = 100)	Total O1 (n = 50)	Total O2 (n = 50)	Difference O1 - O2 (n = 50)	temporal + parietal bone (n = 64)	Occipital bone (n = 16)	Basi-sphenoidal bone (n = 20)
Median	1.57	1.58	1.44	0.32	1.51	2.04	1.56
Minimum	0.42	0.42	0.42	0	0.42	0.68	0.42
Maximum	5.14	4.8	5.14	1.09	3.13	2.97	5.14

O – Observer

## Discussion

The application accuracy of 1.57 mm (range 0.42–5.14 mm) established in this study is in a similar range as previously reported with different veterinary optical neuronavigation devices, in which mean values of 1.79 to 4.3 mm and median target point deviations of 0.79 to 3.53 mm were calculated [1, 2, 5, 7, 11]. According to a review article, neuronavigation systems in human medicine operate with a mean application accuracy of 1.5 to 5.4 mm when used on clinical patients for brain biopsies, electrode placement for deep brain stimulation or ventricle catheter placement [12]. A human medical study that tested the accuracy of the navigation panel unit (STORZ) for the use in ear, nose and throat (ENT) surgery on a phantom determined an accuracy of 1.44 mm (SD 0.18 mm) in the surgical setup and of 0.63 mm (SD 0.07 mm) under ideal conditions [13].

The application accuracy, in contrast to the technical accuracy, refers to the overall error in clinical use and includes all errors associated with registration, imaging and fusion of imaging data sets, target point determination, as well as measurement inaccuracies of the instruments and the so-called “surgeon error”. Potential sources of error when using neuronavigation devices include incorrectly or loosely placed markers, use of too few markers, faulty image data import, poor fixation of the patient tracker or an incorrect registration of the patient before the operation (image-to-patient-registration). To minimize the risk of increased target error, the used neuronavigation system had a pre-set limit of 0.5 mm for the registration process. The device allows switching in the operating mode only if the patient itself is conscientiously registered with a registration error below this limit. The patient registration error for the registration of the five dog heads used in this study was 0.07–0.21 mm (maximum registration error: 0.1–0.32 mm) and thus had only a minor influence on the total error.

In terms of study design, only one veterinary study is approximately comparable to the design described here, because the study also determined the accuracy of a neuronavigation system by determining target point deviations at target points on the bony surface of the skull rather than target points within the brain parenchyma. The study determined the application accuracy of the Brainsight neuronavigation device on the bony surface for the approach to the pituitary gland [5]. In the study a median target point error of 3.533 mm with a range of 2.013 to 4.745 mm was detected [5]. In the present study the median target point error in the region of the access to the pituitary gland was 1.56 mm with a range of 0.42 to 5.14 mm and is therefore in a similar range. The overall largest target point deviation of 5.14 mm was created in the first dog when accessing the pituitary gland. No transphenoidal surgery is performed in the authors’ institution, but for better comparability, this region should be included in the study design. That is why the surgeon performing the procedure initially had difficulties with the surgical access and the handling of the devices for this region. However, a steep learning curve was observed in this region. A similar steep clinical learning curve when using neuronavigation devices, has previously been described in other studies [2, 14]. Furthermore, it is known that the experience of the surgeon in handling optical neuronavigation devices plays a role in using them as accurately as possible [11]. Because of the need for a training before using a neuronavigation device the first time in clinical patients, a three-dimensional dog brain phantom for stereotactic brain biopsy training should be used [14].

In addition to optical neuronavigation devices, which are also known as frameless stereotaxy, various frame-based stereotactic systems are used for brain surgery, primarily for selective interventions in the brain such as brain biopsies or the implantation of electrodes for deep brain stimulation. In human medicine, there is no consensus about the fact which system is more accurate and thus better for brain surgery [15]. For a long time, it was assumed that frame-based stereotaxy is superior to frameless sterotaxy, which is why frame-based systems are recommended as gold standard for deep brain surgery and biopsy of small intracranial lesions [16, 17]. Some systematic reviews and meta-analysis determine no difference between frame-based and frameless stereotactic brain biopsy procedure in terms of diagnostic yield, morbidity and mortality [15, 17]. However, frameless stereotactic brain biopsies have shorter procedural times [15, 18]. One meta-analysis determined an increased risk of asymptomatic hemorrhage using frameless stereotaxy for brain biopsies [17].

In veterinary medicine various frame-based systems are available for stereotactic interventions. The majority of those are systems from human medicine that have been modified for veterinary use, but there are also custom-made designs and 3D printing-based patient-specific framework systems available [19–31]. Applications accuracies of 0.9 and 3.5 mm and median values of 0.83 and 2.7 mm have been described for veterinary frame-based stereotactic systems [20, 21, 24, 26, 27, 29, 30]. It is currently impossible to recommend the use of either frame-based or frameless stereotactic systems for brain biopsies in veterinary neurology since reported data regarding application accuracy, morbidity, mortality and duration of the procedure are very inhomogeneous [32]. However, there are some advantages and disadvantages of both systems, which should be explained briefly:

Frameless neuronavigation systems are more flexible for the surgeon and the patient [10, 17, 33]. Especially the surgeon benefits from the possibility of changing the positioning intraoperatively, which is not possible with frame-based systems. In human medicine the comfort for the patient without a frame is also an important additional point [17, 34].

The virtual reality made by the neuronavigation systems facilitates 3D perception and orientation for the surgeon during the operation, which is particularly important in deep brain surgery. With the use of neuronavigation systems a better planning of the operation, for example in case of brain tumor removal, is often possible [35]. This results in smaller surgical access to the brain, minimized surgical trauma and shorter procedure times [15, 34, 35]. When using neuronavigation systems, it is easier to deviate from the original surgical plan and, for example, take an additional biopsy from a different depth or with a slightly different trajectory to the desired target region [8]. This is significantly more challenging with frame-based systems. Furthermore, neuronavigation systems can be used based on computed tomography images or based on fused CT and MR images, depending on the characteristics of the intracranial lesion or brain tumor.

A disadvantage of using neuronavigation systems that should not be neglected, is the fact that the images used for neuronavigation represent the brain anatomy before a craniotomy was performed. Once the skull has been opened a so-called “brain shift” may occur. Brain shift is a complex process and not easy to predict. Many factors must be considered such as size and type of tumor or lesion, perilesional edema, brain perfusion, anesthetic medication, gravity, osmotic pressure, patient positioning and loss of tissue and cerebrospinal fluid [36, 37]. The surgeon must be aware of the risk of brain shift without being able to determine the extend intraoperatively. That is why in human medicine, the use of a neuronavigation system is often combined with intraoperative MRI or ultrasound [38, 39].

Furthermore, the risk for tremor or deviation from trajectory to the target point is increased when using neuronavigation systems compared to frame-based systems due to more complex hand-eye coordination being necessary [17]. However, this risk could be minimized by training before using the neuronavigation system as already explained above.

Another disadvantage of modern neuronavigation systems are the high purchase costs. These are significantly lower in the case of custom-made frame-based systems and especially in the case of use of 3D-based patient-specific stereotactic frames [30, 33].

There are some limitations of the study presented here. The aim of the study was to determine the accuracy of the device as support for choosing the optimal craniotomy borders for neurosurgical intervention. That is why just the accuracy of the entry points at the skull surface was evaluated. One major limitation of the study is, that these results cannot be extrapolated for the target accuracy in deep brain surgery, because the target point deviation in the brain tissue could be much larger. Another limitation is that only meso- and dolichocephalic dogs weighing more than 10 kg were used. That is why the results are not readily transferable to brachycephalic and toy breeds. However, the optical neuronavigation system described here was used for brain biopsies in brachycephalic and toy breeds obtaining diagnostic samples from the brain lesions [8]. Another limitation of the study is a certain subjectivity in the determination of the coordinates of the drill holes. That is why this step was carried out independently of each other by two observers, but the differences between the investigators were not significant.

## Conclusions

The application accuracy of 1.57 mm (range 0.42–5.14 mm) of the optical neuronavigation system described in this study is in a similar range as results for other veterinary optical neuronavigation devices and is therefore recommended for clinical use as guide for craniotomies in dogs. Optical neuronavigation systems create a virtual reality image of the brain allowing the surgeon to track instruments in real time and can therefore help minimize the surgical access to the brain and surgical trauma and accordingly reduce operation time.

## Data Availability

The datasets generated and analyzed during the study are available from the corresponding author on reasonable request.

## Declarations

### Prior publication

Parts of the results have been presented at the 34rd ESVN-ECVN Symposium 2022 as well as at the 68th DVG Vet-Congress in Berlin in 2022.
